# Acellular Bone Marrow Extracts Significantly Enhance Engraftment Levels of Human Hematopoietic Stem Cells in Mouse Xeno-Transplantation Models

**DOI:** 10.1371/journal.pone.0040140

**Published:** 2012-07-02

**Authors:** Kazem Zibara, Rima Hamdan, Leila Dib, Steen Sindet-Pedersen, Mohamed Kharfan-Dabaja, Ali Bazarbachi, Marwan El-Sabban

**Affiliations:** 1 Department of Biology, Doctoral School of Science and Technology (DSST), Lebanese University, Beirut, Lebanon; 2 Department of Anatomy, Cell Biology and Physiological Sciences, Faculty of Medicine, American University of Beirut, Beirut, Lebanon; 3 Department of Oral and Maxillofacial Surgery, Nicolas & Asp University College, Dubai Healthcare City, Dubai, United Arab Emirates; 4 Department of Internal Medicine, American University of Beirut, Beirut, Lebanon; University of Sao Paulo - USP, Brazil

## Abstract

Hematopoietic stem cells (HSC) derived from cord blood (CB), bone marrow (BM), or mobilized peripheral blood (PBSC) can differentiate into multiple lineages such as lymphoid, myeloid, erythroid cells and platelets. The local microenvironment is critical to the differentiation of HSCs and to the preservation of their phenotype *in vivo*. This microenvironment comprises a physical support supplied by the organ matrix as well as tissue specific cytokines, chemokines and growth factors. We investigated the effects of acellular bovine bone marrow extracts (BME) on HSC *in vitro* and *in vivo*. We observed a significant increase in the number of myeloid and erythroid colonies in CB mononuclear cells (MNC) or CB CD34+ cells cultured in methylcellulose media supplemented with BME. Similarly, in xeno-transplantation experiments, pretreatment with BME during *ex-vivo* culture of HSCs induced a significant increase in HSC engraftment *in vivo*. Indeed, we observed both an increase in the number of differentiated myeloid, lymphoid and erythroid cells and an acceleration of engraftment. These results were obtained using CB MNCs, BM MNCs or CD34^+^ cells, transplanted in immuno-compromised mice (NOD/SCID or NSG). These findings establish the basis for exploring the use of BME in the expansion of CB HSC prior to HSC Transplantation. This study stresses the importance of the mechanical structure and soluble mediators present in the surrounding niche for the proper activity and differentiation of stem cells.

## Introduction

Hematopoietic stem cell transplantation (HSCT) is an established treatment for patients with various hematological malignancies, with an increasing frequency of procedures worldwide [1]. The source of hematopoietic stem cells (HSC) used for HSCT includes bone marrow (BM), cord blood (CB), or mobilized peripheral blood (PBSC).

In the allogeneic HSCT setting, CB represents an ideal cell source as it is readily available in large numbers and is associated with a lower risk of graft-versus-host disease (GvHD) [2]. The major drawback for the use of CB is the relatively limited number of HSC limiting its use to pediatric patients less than 40 kg of bodyweight when using a single cord unit [3]. To overcome this limitation, pooling of double cord is an alternative in adult patients [4]. This approach, however, has several limitations including difficulty in finding compatible CB units. As a result, *ex-vivo* expansion of the pool of HSCs from a single CB unit represents the ideal approach to expand applicability of CB for HSCT. It is important to note that the use of double CB units leads to a similar rate of GvHD compared to grafts from healthy donors [5].

In the autologous HSCT setting, the major source for HSC is mobilized PBSC that are collected by aphaeresis and cryopreserved [6]. These cells contain not only stem cells but also more mature committed progenitors and precursors that participate to early engraftment. *Ex-vivo* expansion of the pool of committed progenitors and precursors prior to the administration of PBSC could result in faster engraftment, hence reducing length of hospital stay and potentially result in cost savings.

The *in vivo* local microenvironment is critical to support the differentiation of stem cells or to sustain the phenotype of the stem cell-derived *in vitro* differentiated cells [7], [8]. This local microenvironment comprises a physical support supplied by the organ matrix as well as tissue specific factors. In previous studies [9], we demonstrated that acellular organ extracts induce tissue specific differentiation of BM derived mesenchymal stem cells (MSC). We first showed that acellular bovine or equine bone extracts induce osteoblatic differentiation of human bone marrow derived MSC. We also demonstrated that acellular cartilage extracts (derived from meniscus and joint cartilage) induce chondrocytic differentiation of BM derived MSC (unpublished data).

In this study, we investigated the effects of acellular bovine BME on hematopoietic progenitor development in methylcellulose assays and in HSC engraftment in xeno-transplantation models. We show that BME induce and stimulate the growth of myeloid and erythroid colonies in methylcellulose cultures of CB or BM mononuclear cells or CD34 positive cells. Importantly, we also demonstrate that *ex-vivo* pretreatment of HSC with BME significantly improve and accelerate their *in vivo* engraftment in xeno-transplantation models in NOD/SCID and NSG mice. These results support the use of BME in the expansion of CB and PBSC hematopoietic progenitors and/or HSC prior to HSCT.

## Materials and Methods

### Ethics Statement

Consent forms detailing the use of human cord blood (CB) and bone marrow (BM) in our experimental protocol were approved by the American University of Beirut Medical Center (AUB-MC) Institutional Review Board (IRB). All animal experiments were approved by the AUB Institutional Animal Care and Use Committee **(**IACUC**)**. Animals were handled under pathogen-free sterile conditions, maintained under micro-isolators, and fed sterile food. Finally, this study was performed in accordance with the principles of the declaration of Helsinki.

### Human Cord Blood and Bone Marrow Cells

Samples of CB were obtained from discarded placental and umbilical tissues, after informed consent from delivering mothers attending the AUB-MC. BM cells were collected after informed consent of patients attending the hospital for BM aspirations. Bone marrow samples were derived from patients diagnosed with Hodgkin disease or Non-Hodgkin Lymphomas, who had routine BM assessment as staging for their disease and who were found to have a normal bone marrow. CB and BM were collected in tubes containing EDTA, sodium citrate or heparin, and were processed within 24 hours.

### Purification of MNCs by Ficoll

Fresh samples of CB were diluted 1∶3 in Iscove’s modified Dulbecco’s medium (IMDM, GIBCO-BRL, Life Technologies, Saint Aubin, France) containing 10% fetal bovine serum (FBS, Life Technologies). Samples were enriched for mononuclear cells (MNC) by centrifugation on Ficoll/Hypaque (GE Healthcare Life Sciences, Uppsala, Sweden). This step of density gradient centrifugation would allow the purification of MNCs from red blood cells and neutrophils. These cells were either used immediately for the experiments described below or cryopreserved in 10% dimethylsulfoxide (DMSO, Sigma GmbH, Roedermark, Germany) in FBS. Later, cryopreserved cells were thawed at 37°C, washed in phosphate-buffered saline (PBS), and resuspended in medium for the specific experiment. After thawing, cell viability was monitored by Trypan blue dye exclusion.

### Isolation of CD34^+^ Cell Populations

Enrichment of HSCs was obtained by isolation of the CD34^+^ fraction from BM cells collected from free of disease patients after informed consent. MS columns and µMACS magnetic beads (Miltenyi Biotec, Surrey, UK) or EasySep columns (Stem Cell Technologies, Vancouver, Canada) were used for this purpose. Using a magnetic separation procedure, the CD34^+^ fraction of HSCs was positively selected by labeling with a CD34 antibody that is anchored to magnetic beads. As a result, about one million CD34 positively selected cells were isolated from 100 million BM MNCs. Purity of the isolated fraction was measured by flow cytometry and was always determined to be greater than 90%. Data acquisition and analysis was performed with CellQuest or Diva softwares from Becton Dickinson (BD, New Jersey, USA) or with FlowJo software (Treestar, Oregon, USA).

### Bovine Bone Marrow Extracts (BME)

Extracts are sterile acellular lyophilizates from bovine BM matrix and were supplied by Ossacur AG, (Oberstenfeld, Germany). This new technology is based on the extraction of extracellular matrix proteins from animal tissue and contains a mixture of all growth factors and cytokines within the matrix. Briefly, extracts were prepared, aseptically, from bone marrow of disease-free, young (<12 months old) calves of closed herds. Bone marrows were pulverized and delipidated with acetone for 60 min at 4°C. The resulting particles were demineralized in 0.6 N hydrochloric acid for 60 min at 4°C. Particles were then washed in deionized water, extracted with 4 M guanidine hydrochloride and ultrafiltered using 3 K nominal molecular cut-off membranes. Extracts were then reconstituted in water from the initial stock solution in order to have a final concentration of 0.0138 mg/ml. This concentration was selected based on previous liquid culture experiments, which showed optimum differentiation results (data not shown).

### Culturing and Treatment of MNCs and CD34^+^ Cells

MNCs were cultured, in 25 cm^2^ Falcon dishes, in Iscove’s modified Dublecco’s medium (IMDM, GIBCO-BRL, Life Technologies,) supplemented with 10% fetal bovine serum (FBS) for 7 days. On the other hand, about 20,000 CD34^+^ cells were seeded in 6-well plates and suspended at 10^5^ cells/mL in serum-free StemSpan H3000 culture medium (Stem Cell Technologies). The CD34+ cells were supplemented with SCF (25 ng/mL) and G-CSF (10 ng/mL) recombinant human factors for 13 days (Invitrogen, Life Technologies). Cells were treated with BME, diluted with culture medium to have a final concentration of 0.0138 mg/ml. Control cells were left un-treated, i.e. without the addition of BME. Cell suspensions were incubated at 37°C in a 5%CO2/95% air atmosphere, after which they were collected, washed in IMDM, counted by Trypan blue exclusion, analyzed for progenitor and stem cells, and engrafted into NOD-SCID or NSG mice.

### Colony Forming Cell (CFC) Assay

CD34^+^ cells from each CB sample were suspended in IMDM/10% FBS and plated on methylcellulose-based media (HSC002, HSC003, and HSC004, R & D systems, Lille, France), with or without the addition of BME. The human methylcellulose base media HSC002 contains 1.3% methylcellulose, 25% FBS, 2% BSA, 2 mM L-glutamine, and 5×10^−5 ^M beta-mercaptoethanol. The human methylcellulose complete media HSC003 contains the basic HSC002 media and is supplemented by rh-SCF (50 ng/mL), rh-GM-CSF (10 ng/mL), rh-IL-3 (10 ng/mL), and rh-Epo (3 U/mL). The human methylcellulose complete media HSC004 is similar to HSC003 media but without Epo. Cells were plated, in duplicate, at a final concentration of 100 cells/9.6 cm^2^ dish. Colonies consisting of CFU-GM (Granulocyte Macrophage), CFU-E/BFU-E (Erythroid), and CFU-GEMM (Granulocyte Erythroid Macrophage Megakaryocyte) were counted at days 14–16 under an inverted microscope.

### Mice

NOD/SCID (NOD.CB17-*Prkdc* ^scid^/J) and NSG mice (NOD.Cg-*Prkdc* ^scid^ *Il2rg* ^tm1Wjl^/SzJ) were obtained from the Jackson Laboratory (Maine, USA).

### Conditioning Regimen

We used the alkylating agent, busulfan (Busilvex, Pierre Fabre, Boulogne, France), as conditioning regimen for transplantation. Busulfan is a cell cycle non-specific drug that functions similarly to total body irradiation (TBI). It provides equivalent sensitivity to the standard irradiation regimen conditioning protocol in immune-compromised mice, as demonstrated by HSC detection, evaluated by limiting dilution analysis of SRC [10]. Mice aged 6–10 weeks received intra-peritoneal (*ip)* injection of Busulfan at a dose of 22 mg/kg, for 3 consecutive days before transplantation. HSCs are then injected intravenously.

### Transplantation of Isolated Cells into NOD/SCID or NSG Mice

Purified cell populations (MNCs or CD34^+^), at the indicated dose, were transplanted by tail-vein intravenous (*iv)* injection into Busilvex treated mice according to standard protocols. Briefly, human cells were suspended in 250 µl IMDM tissue culture medium (Sigma) containing 1% human serum albumin (HSA, ZLB Behring GmbH, Marburg, Germany). Groups of five mice each, received a dose of viable human CB MNCs (1.5×10^6^, 3×10^6^, or 4×10^6^ cells/mouse). If purified CD34^+^ cells were used, mice were transplanted with 50×10^3^ cells/mouse. The effect of BME on engraftment was studied by culturing the same amount of cells with or without BME for 7 days.

### Mice Sacrifice and Collection of Samples

Mice were sacrificed at 3 or 6 weeks after transplantation. Mice were euthanized by Isoflurane inhalation (Abbot Laboratories, Kent, UK) then cervical dislocation. BM was collected from the long bones of the two posterior limbs (femurs, tibiae, and iliac crests) by flushing with IMDM containing 10% FBS. Single cell suspensions were prepared by drawing the BM cells through a 27-gauge needle, then expelling them back through the needle and through a nylon mesh (70 µ strainers, BD).

### Flow Cytometry and Assessment of Engraftment

Collected BM samples from NOD/SCID or NSG mice were stained with various human specific monoclonal antibodies and analyzed by flow cytometry, after 3 or 6 weeks of transplantation. Briefly, approximately 10^5^ cells were suspended in 50 µl of PBS/5% FCS/5% human serum for 30 min at 4°C, in order to block Fc receptors. Cells were then washed in PBS containing 10%FBS, and incubated with monoclonal antibodies at a concentration of 5 µg/ml for 30 min at 4°C. Cells were stained with human specific FITC-conjugated anti-CD33, PE-conjugated anti-CD19 and Phycoerythrin-Cyanin 5 (PE-Cy5/PC5)-conjugated anti-CD45. Cells were also stained with human specific FITC-conjugated anti-CD235a (Glycophorin A), PE-conjugated anti-CD36 and Peridinin-Chlorophyll-Protein complex (PerCP)-conjugated anti-CD45 antibodies. Following staining, cells were washed in PBS/5% FBS and analyzed on a flow cytometer (BD FACSCalibur or BD FACSCanto, New Jersey, USA). Gates were set up to exclude nonviable cells and debris. For each mouse analyzed, an aliquot of cells was also stained with isotype control mouse immunoglobulin G (IgG) conjugated to FITC, PE, PC5 or PerCP (mouse IgG1, IgG2a, IgG2b, or IgM; Becton-Dickinson). BM cells from an untransplanted mouse were stained in parallel as a negative control. Fluorescence levels excluding greater than 99% of the cells in these negative controls were considered to be positive and specific for human staining. About 50,000 events were collected for data analysis. Data acquisition was done using SciQuest or Diva softwares from Becton Dickinson while data analysis was performed on flowJo (Treestar, USA). The level of engraftment (i.e. % of human cells) in the BM of NOD/SCID or NSG mice was then measured by adding the engraftment levels from the human leukocytes population (CD45^+^ cells) in addition to immature erythroblasts (CD45^−^CD36^+^CD235a^+^). Furthermore, multi-lineage differentiation of human cells in murine BM was also determined. Indeed, human CD45^+^ cells were further gated based on characteristic forward and side scatter properties into lymphoid and myeloid populations. Multi-lineage engraftment is defined by the presence of separate CD45^+^CD33^+^ (myeloid) and CD45^+^CD19^+^ (lymphoid) populations with appropriate scatter characteristics. Moreover, erythroid populations made up of immature erythroblasts (CD45^−^CD36^+^CD235a^+^) or mature RBC’s (CD45^−^CD36^−^CD35a^+^) were also determined as explained in previous publications [10], [11]. Finally, in order to confirm human engraftment, murine BM cells were also stained with murine specific FITC-conjugated anti-CD45. The engraftment pattern of mice transplanted with purified CD34^+^ cells was similar to what is observed after transplantation of unsorted CB cells. CD45 and CD34 antibodies were purchased from Becton Dickinson (NJ, USA). All other antibodies were from Beckman Coulter (California, USA). It’s important to note that myeloid and B lymphoid reconstitution can be easily attained in NOD-SCID and NSG mice by the transplantation of human HSCs. Several reports have suggested that T cells may be able to develop in the thymus of NOD-SCID mice transplanted with human CD34 cells. In particular, van der Loo *et al.* succeeded in repopulating a mouse thymus with human cells by administering G-CSF and stem cell factor. However, most of the T cells were double positive (DP) cells, and a functional analysis of these T cells has not been completed [12]. Recently, Yahata et al reported a new model for the study of T-Lymphocytes in the NOD/Shi-scid, IL2-Receptor gamma null mouse background [13].

### Statistical Analysis

Results are expressed as individual data or as the mean±SD. Statistical comparisons were performed using the Student’s t-test in order to determine statistical significance. Paired t-test was used for methylcellulose experiments. The p value was determined and values for p<0.05 or p<0.001 were considered significant. The Microsoft Excel and SPSS Packages were used to perform statistical analysis.

## Results

### Bone Marrow Extracts Enhance Hematopoietic Colony Formation

**Figure 1:**
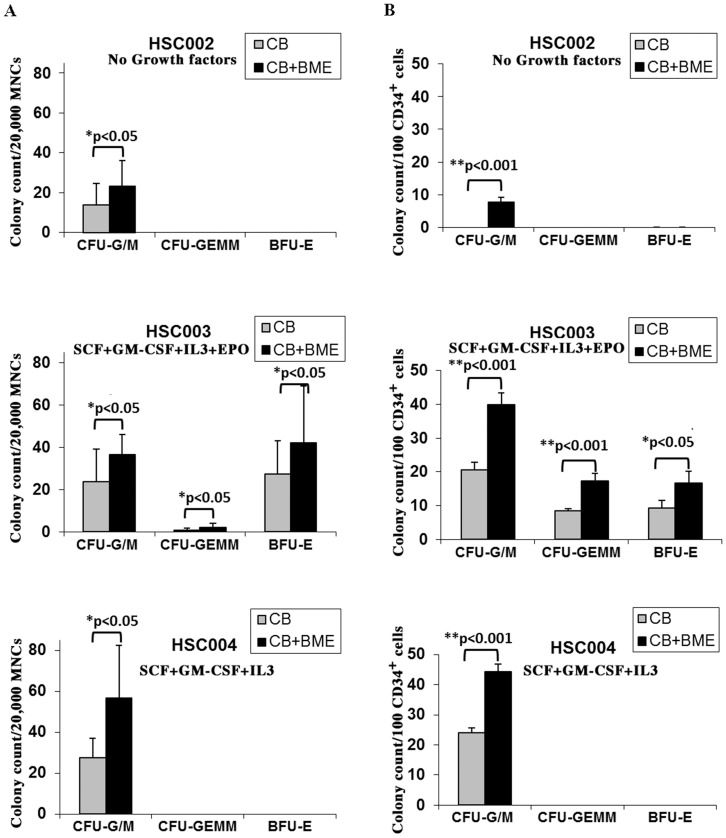
Bone marrow extracts enhance hematopoietic colony formation. **A**) About 20,000 CB MNCs from each patient were plated on methylcellulose-based media (HSC002, HSC003, and HSC004), in the presence or absence of BME (CB+BME or CB, respectively). Methylcellulose media contained no growth factors (HSC002), SCF+GM-CSF+IL3+Epo (HSC003), or SCF+GM-CSF+IL3 without Epo (HSC004). Colony forming units (CFU) consisting of CFU-G/M (Granulocyte or Macrophage or both), CFU-E/BFU-E (Erythroid), and CFU-GEMM (Granulocyte Erythroid Macrophage Megakaryocyte) were counted at day 16 using an inverted microscope. Data represents an average of 6 different samples. **B**) A total of 100 CB CD34+ cells from each patient were plated on methylcellulose media (HSC002, HSC003, and HSC004), in the presence or absence of BME (CB+BME or CB, respectively) as described above. Data represents an average of 4 samples used in the study. Paired TTest was used for statistical significance (*: p<0.05, **: p<0.001).

We first investigated the effect of acellular BME on the proliferation and differentiation of CB hematopoietic progenitors using colony-forming assays in methylcellulose. A total of 20,000 CB MNCs from each patient (n = 6) were plated on different types of methylcellulose-based media (HSC002, HSC003, and HSC004), in the presence or absence of BME (CB+BME or CB, respectively). In HSC002 media, which does not contain any added growth factors, the addition of BME increased the number of CFU-G/M colonies by 1.7-fold (Figure 1A, upper panel). In HSC003 media, which contains SCF, GM-CSF, IL3 and Epo, the addition of BME resulted in a significant increase of approximately 1.5 fold in the number of myeloid colonies (CFU-G/M), erythroid colonies (BFU-E) and mixed colonies (CFU-GEMM) (p<0.05 for all colony types) (Figure 1A, middle panel). Finally, in HSC004 which contains SCF, GM-CSF and IL3, BME increased the number of myeloid colonies by at least 2-fold (Figure 1A, lower panel). Similar results were obtained when positively selected CB CD34^+^ cells were used. Indeed, when 100 CD34^+^ cells from each CB sample (n = 4), were plated on the basic methylcellulose media without any added factors (HSC002), myeloid colonies (CFU-G/M) appeared only in the presence of BME (Figure 1B, upper panel). Moreover, addition of BME resulted in a significant increase of myeloid colonies (2 fold) in HSC003 and HSC004 (p<0.001), of erythroid colonies in HSC003 (p<0.001), and of GEMM in HSC003 (p<0.05) (Figure 1B, middle and lower panels). Since HSC002 and HSC004 media presented neither CFU-GEMM nor BFU-E (erythroid) colonies, we may speculate that BME do not contain any EPO activity. Overall, BME increased the number of myeloid and erythroid colonies in methylcellulose culture from both CB MNC and CB CD34^+^ cells.

### Bone Marrow Extracts Enhance Human Cord Blood Derived Mononuclear Cell Engraftment in Immune-deficient Mice

The current models for assessment of transplantable human HSCs are based on the use of immune-deficient mice such as NOD/SCID [14] (NOD.CB17-*Prkdc* ^scid^/J) and NSG [15] (NOD.Cg-*Prkdc* ^scid^ *Il2rg* ^tm1Wjl^/SzJ) mice strains. NOD/SCID mice are characterized by low NK cell activity [16], in addition to B-cell, T-cell, and complement defects. NSG mice represent a more permissive xeno-transplantation model generated from NOD/SCID and the IL-2 receptor gamma c (CD132) knock-out mice and characterized by complete lack of NK activity.

**Figure 2:**
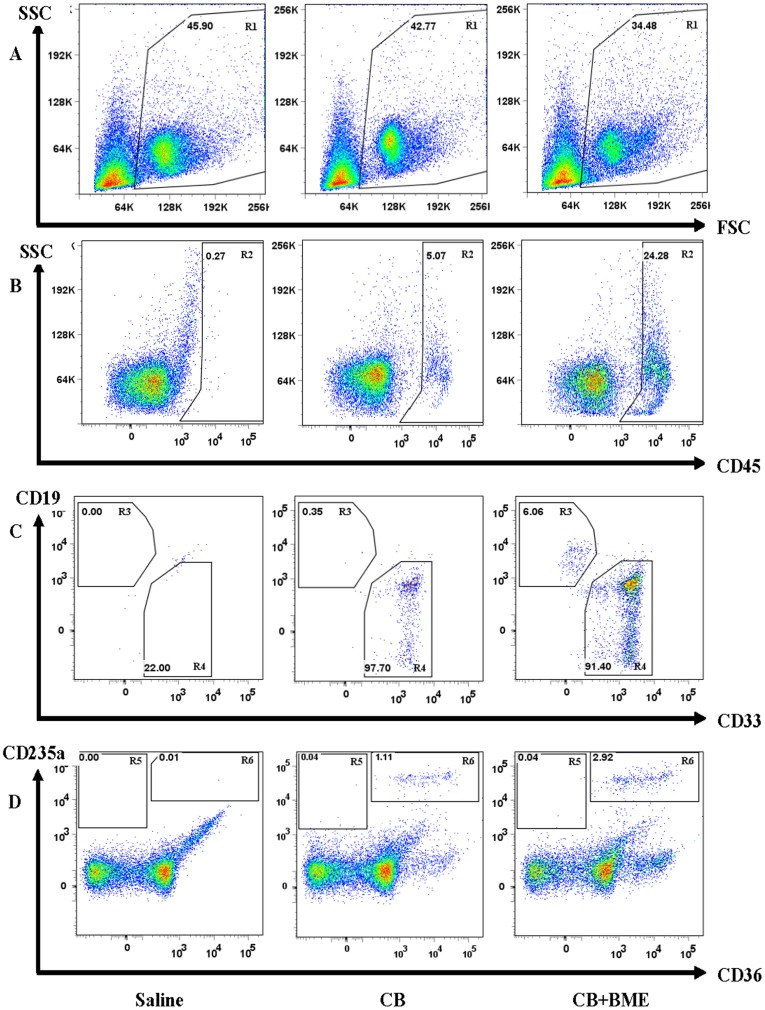
Multilineage engraftment of human CB MNC in NSG mice. In this study, NOD/SCID or NSG mice received *iv* busulfan conditioning followed by *iv* injection of CB MNC or CD34^+^ cells that were previously cultured *in vitro* for 7 days, in the presence or absence of BME. This figure represents NSG mice which received *iv* injection of 3×10^6^ CB MNC according to the same protocol. Mice were sacrificed and bone marrow cells were harvested from femurs, tibia and pelvis and examined for multilineage engraftment by flow cytometry according to the following gating strategy: **A**) Live cells were first gated using forward scatter versus side scatter plots (R1 region). The three plots represent mice injected with saline (negative control), CB, or CB+BME; respectively. **B**) Human leucocytes were then gated using human CD45 staining (pan-leukocyte marker, R2 region). **C**) From CD45^+^ gate (R2 region), cells were then examined for multi-lineage engraftment defined by the presence of separate lymphoid (CD45^+^CD19^+^, R3 region) and myeloid (CD45^+^CD33^+^, R4 region) populations. **D**) The population of CD45^−^ cells were gated in order to determine the erythroid populations. Indeed, erythroid populations made up of mature RBCs (CD45^−^CD36^−^CD235a^+^, R5 region) or immature erythroblasts (CD45^−^CD36^+^CD235a^+^, R6 region) were also determined. It’s important to note that the CD36^+^CD235a^−^ population, which is not present in the controls, does not represent immature erythroblasts but are CD45+ mature cells. The percent engraftment was defined as the total number of leucocytes and immature erythroblasts (CD45^+^ and CD45^−^CD36^+^CD235a^+^ cells).

A representative example of the data in an NSG mouse is shown in Figure 2. At six weeks, the percentage of engraftment (CD45^+^ fraction) increased from 5% in an NSG mouse injected with untreated CB MNC cells to about 24% in a mouse injected with cells treated with BME. This increase in engraftment was mainly due to an increase in the number of myeloid cells (CD33^+^, R4 region), which represented more than 90% of engrafted cells. Finally, the percentage of engraftment due to immature erythroblasts increased from around 1.11% in the mouse injected with untreated CB MNC cells to around 2.92% in the mouse injected with CB MNC treated with BME (Figure 2).

**Figure 3:**
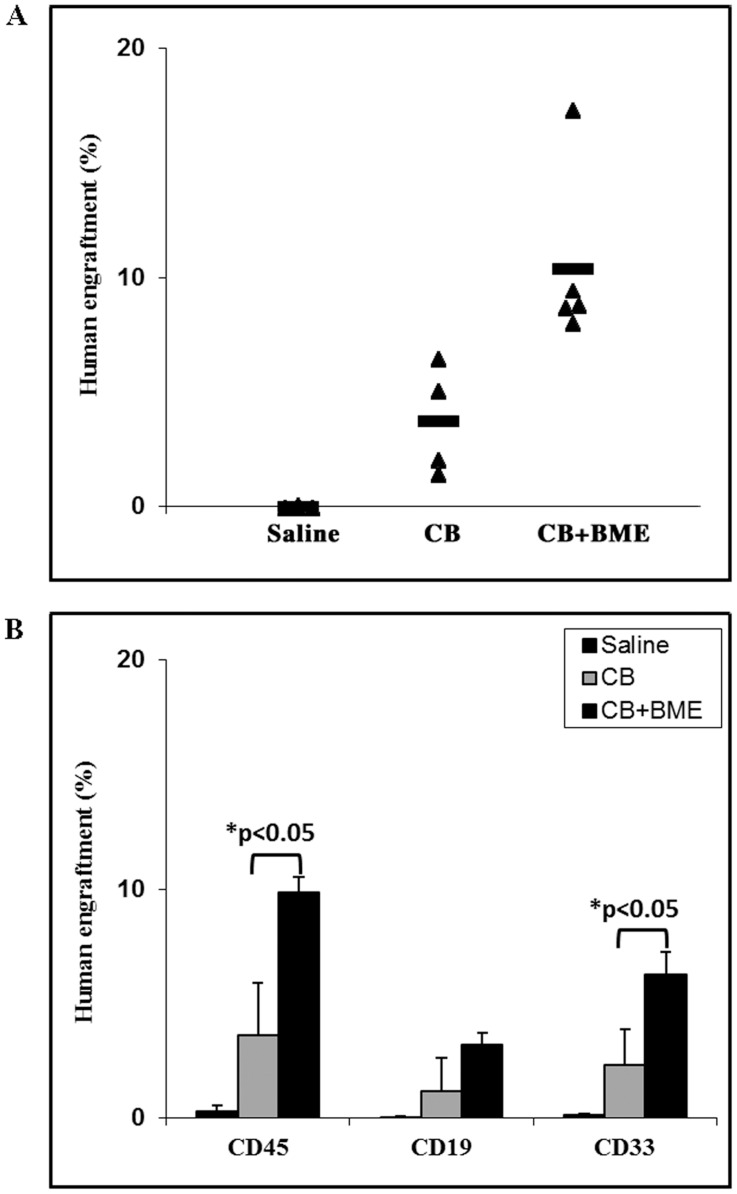
Bone marrow extracts enhance human cord blood derived mononuclear cell engraftment in NOD/SCID mice. Two groups of NOD/SCID mice (n = 5 each) received 3×10^6^ CB MNCs previously cultured for 7 days, in the presence or absence of BME. A third group (n = 5) served as a negative control and were injected with saline. Mice were sacrificed six weeks after transplantation. **A**) The percentage of engraftment was determined using the human pan leukocyte CD45 marker. **B**) The contribution of myeloid and lymphoid populations to total human leukocyte engraftment was determined using CD33, CD19 and CD45 markers, respectively. *: p<0.05.

**Figure 4:**
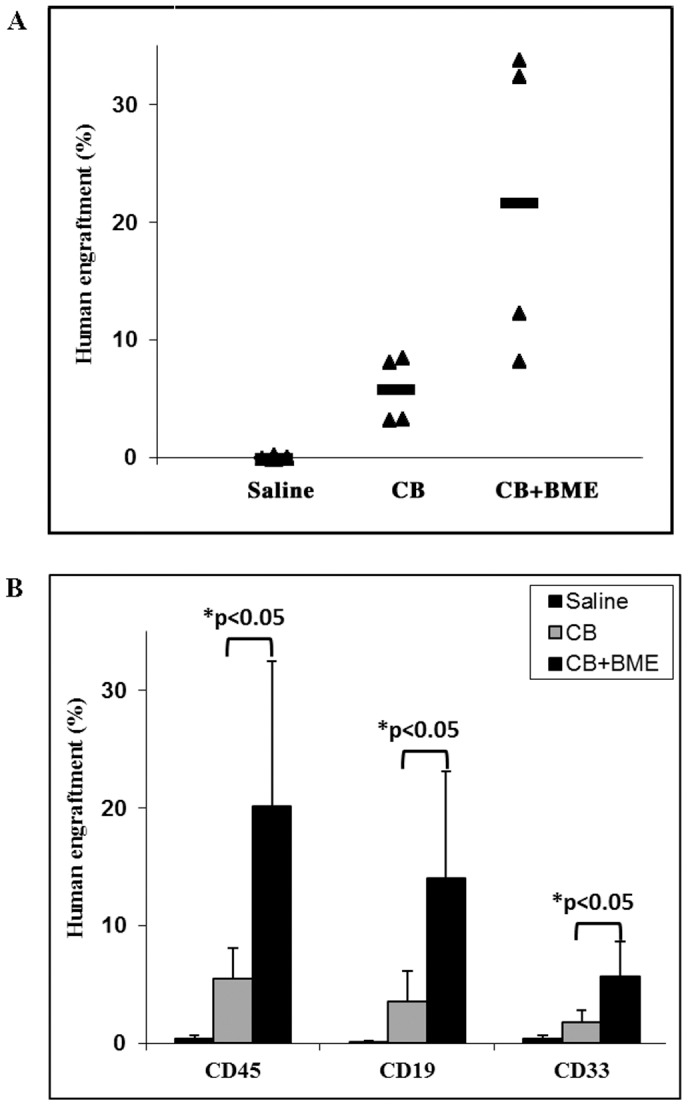
Bone marrow extracts enhance human cord blood derived mononuclear cell engraftment in NSG mice. Two groups of NSG mice (n = 4 each) received 1.5×10^6^ CB MNCs previously cultured for 7 days, in the presence or absence of BME. A third group (n = 5) served as a negative control and was injected with saline. Mice were sacrificed six weeks after transplantation. **A**) The percentage of engraftment was determined using the human pan leukocyte CD45 marker. **B**) The contribution of myeloid and lymphoid populations to total human leukocyte engraftment was determined using CD33, CD19 and CD45 markers, respectively. *: p<0.05.

Two groups of NOD/SCID mice (n = 5 each) received CB MNCs that were put in culture for 7 days, in the presence or absence of BME. A third group (n = 5) served as a negative control and was injected a saline solution. Mice were sacrificed six weeks after transplantation. The percentage of engraftment, as determined by the human pan leucocytic CD45 marker, significantly increased from 3% in the mice that have received untreated CB MNC cells to more than 10% in the mice which received CB MNC cells cultured in the presence of BME (p<0.05) (Figure 3, upper panel). When subpopulations were examined, the increase in engraftment was significant for the myeloid populations (p<0.05) but not for lymphoid cells (Figure 3, lower panel). Similar results were obtained in NSG mice (n = 4 for each group) which received CB MNCs that were put in culture for 7 days, in the presence or absence of BME. The percentage of engraftment, at 6 weeks of transplantation, significantly increased from 5% in the mice that have received cells cultured without BME to 20% in the mice that received cells cultured with BME (p<0.05) (Figure 4, upper panel). When subpopulations were examined, the increase in engraftment was significant for both myeloid (p<0.05) and lymphoid (p<0.05) populations (Figure 4, lower panel).

**Figure 5:**
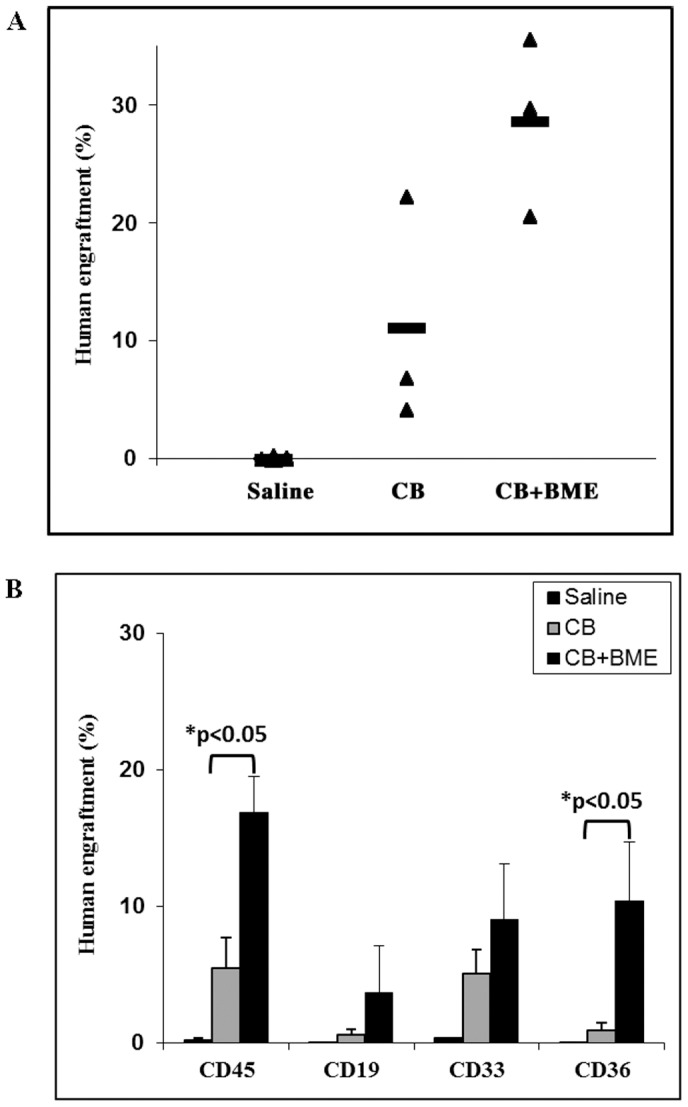
Bone marrow extracts enhance human erythroid engraftment in NSG mice. Two groups of NSG mice (n = 3 each) received 4×10^6^ CB MNCs previously cultured for 7 days, in the presence or absence of BME. A third group (n = 5) served as a negative control and was injected with saline. Mice were sacrificed three weeks after transplantation. **A**) The percentage of engraftment was determined using the human pan leukocyte CD45 marker. **B**) The contribution of myeloid, lymphoid and erythroid populations to total human leukocyte engraftment was determined using CD33, CD19, CD36 and CD45 markers, respectively. *: p<0.05.

In order to assess erythroid engraftment, the percentage of engraftment was measured at three weeks instead of six weeks. The time point at 3 weeks allows seeing engraftment due to immature erythroblasts and RBC’s. NSG mice (n = 3 for each group) received CB MNCs that were put in culture for 7 days, in the presence or absence of BME. In these conditions, the percentage of engraftment significantly increased from 12% in the mice that have received cells cultured without BME to 27% in the mice that received cells cultured with BME (p<0.05) (Figure 5, upper panel). When subpopulations were examined, the increase in engraftment was only significant for immature erythroblasts (CD45^−^CD36^+^, p<0.05) (Figure 5, lower panel).

### Bone Marrow Extracts Enhance Human Bone Marrow Derived CD34 Positive Cells Engraftment in Immune-deficient Mice

**Figure 6:**
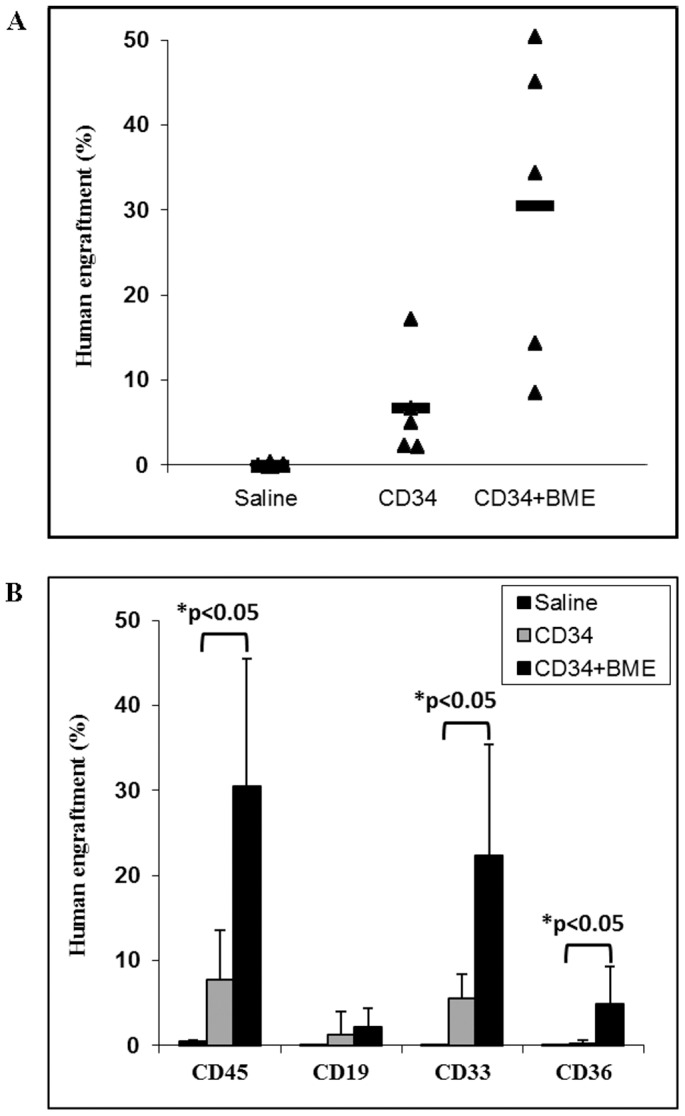
Bone marrow extracts enhance human bone marrow derived CD34 positive cells engraftment in immunodeficient mice. Two groups of NSG mice (n = 5 each) received 50×10^3^ bone marrow derived CD34 positive cells previously cultured for 13 days, in the presence or absence of BME. A third group (n = 5) served as a negative control and was injected with saline. Mice were sacrificed six weeks after transplantation. **A**) The percentage of engraftment was determined using the human pan leukocyte CD45 marker. **B**) The contribution of myeloid, lymphoid and erythroid populations to total human leukocyte engraftment was determined using CD33, CD19, CD36 and CD45 markers, respectively. *: p<0.05.

We then investigated the effect of BME on positively selected CD34 cells derived from normal human bone marrow, using the immune-deficient NSG mice model. Two groups of NSG mice (n = 5 each) received 50×10^3^ CD34 positive cells that were put in culture for 13 days in the presence or absence of BME. A third group (n = 5) served as a negative control and was injected a saline solution. Mice were sacrificed six weeks after transplantation. The percentage of engraftment, as determined by the human pan leucocyte CD45 marker, significantly increased from 8% in the mice that have received untreated CD34 positive cells to more than 31% in the mice that received CD34 positive cells cultured in the presence of BME (p<0.05) (Figure 6, upper panel). When subpopulations were examined, the increase in engraftment was significant for both myeloid (p<0.05) and immature erythroblasts (p<0.05) populations but not for lymphoid cells (Figure 6, lower panel).

## Discussion

Traditionally allogeneic HSCT was offered only to patients with available HLA-matched sibling donors. Introduction of unrelated donors whether using unmanipulated BM, mobilized PBSC or CB has certainly expanded applicability of allogeneic HSCT to patients with various hematologic diseases. Use of umbilical CB cells offers several advantages such as immediate availability, lack of risks to donors, and a lower incidence of GVHD despite the presence of significant HLA disparity. Expanding applicability of allogeneic HSCT using CB cells, particularly to adult patients, has been faced with several challenges including a limited number of available progenitor HSCs in a single CB unit and a delayed hematopoietic cell engraftment. For instance, adult patients undergoing CB allogeneic HSCT have a high likelihood of non-relapse mortality during the early post transplantation phase mainly due to poor recovery of blood counts as a result of the lower number of progenitor HSC compared to BM or PBSC [17]. Transplantation of two partially HLA-matched umbilical CB units is feasible and facilitates performing the procedure in adult patients [18]. However, identification of two compatible CB units is not always possible. Accordingly, *ex vivo* expansion methods to increase cell dose of single CB units represents an unmet need to continue to offer potentially curative allogeneic HSCT to otherwise deadly hematologic and non-hematologic diseases.

Increasing evidence supports a vital role of the local microenvironment *in vivo* in the differentiation of stem cells and for the maintenance of the phenotype of differentiated cells. This local microenvironment comprises a physical support supplied by the organ matrix/marrow as well as tissue specific factors such as cytokines, chemokines and growth factors. It is now clear that ECM-based control of the cell may also occur through multiple physical mechanisms, such as ECM geometry, ECM elasticity, or mechanical signals transmitted from the ECM to the cells [19] in addition to the interactions of ECM ligands with cell surface receptors [20]. We have previously demonstrated [9] that acellular organ “extracts” induce tissue specific differentiation of bone marrow derived mesenchymal stem cells (MSC). Indeed we showed that acellular bovine or equine bone extracts induce osteoblatic differentiation of human BM derived MSC. In addition, cartilage (meniscus and joint) extracts specifically caused the differentiation of MSCs into chondrocytes, with no cross-reactivity to bone. Differentiation markers of either bone- or cartilage- induced differentiation of MSCs were selective and tissue-specific. In this study we postulated that extracts originating from BM should contain factors involved in differentiation and maintenance of HSCs.

We investigated the effects of acellular bovine BME on hematopoietic progenitor development *in vitro*, using methylcellulose colony formation assays and engraftment potential of *ex vivo* treated cells *in vivo*, using immune-deficient mice models. We show that the local microenvironment stimulates the growth of myeloid and erythroid colonies in methylcellulose cultures and is critical for *in vivo* engraftment of HSC in xeno-transplantation models. Indeed, the following several lines of evidence support the above statements: (1) We observed a significant increase of at least 2-fold in the number of myeloid and erythroid colonies in CB MNCs cultured in methylcellulose media supplemented with BME, when compared to cells cultured without BME. (2) A similar increase of about 2-fold in the number of CFU-G/M, CFU-GEMM and BFU-E was also observed when CB CD34^+^ cells were used. (3) Pretreatment with BME during *ex-vivo* culturing of HSCs induces an increase in the level of engraftment *in vivo* in xeno-transplantation mice models (NOD/SCID and NSG). (4) This increase in engraftment levels is supported by an increase in the number of differentiated cells. (5) The effect of BME on multi-lineage engraftment and HSC transplantation *in vivo* was confirmed using CB MNCs, BM MNCs and especially CD34^+^ cells, using both mice models. (6) MNCs and CD34+ cells were cultured for only 1 week or 13d, respectively, instead of the 2–3 weeks used by others. This new method, using BME, with reduced period of culturing time leads to a maximum yield of progenitor, precursor and differentiated cells. (7) Finally, it’s important to note that engraftment in the different conditions tested was due not only to myeloid cells, but also to immature erythroblasts.

In an attempt to elucidate whether the differentiation of stem cells *in vitro* using BME is due to endogenous differentiation factors, different factors were analyzed by Bioscientia GmbH (Ingelheim, Germany). The following results were obtained: Epo (11.3 mIU/mL), IL-2 (2.2 pg/mL), IL-3 (1.3 pg/mL), IL-6 (<1.0 pg/mL), and IL-10 (1.9 pg/mL). Epo concentrations were well below the 20^th^ percentile, which reflects insufficient levels to explain the observed erythropoiesis. Since Epo levels in HSC0003 is in vast excess to that present in our extract, therefore the data suggests that other unknown factors (such as ECM, growth factors, etc…) should be implicated in differentiation.

CB transplants are usually associated with delayed engraftment and consequently increased rates of infectious complications due to the relatively low number of progenitor cells in the graft [21]. Therefore, different methods of *ex vivo* expansion of CB have been used in order to deliver higher cell doses and improved outcomes [22]. In fact, a major goal of *ex vivo* expansion is the production of optimal number of HSCs for transplantation as well as an enough number of specific progenitor cells for rapid recovery from pancytopenia. Different teams have now published promising reports of CB progenitors expanded in liquid cultures [23], [24], [25]. These include the use of different cocktails of cytokines (such as SCF, IL-3, IL-6, G-CSF, TPO, and Flt-3 ligand), marrow-derived mesenchymal cells [26], [27], Notch ligand expansion [28], bioreactors [29], or different stromal [30] co-culture conditions. Usually, *ex vivo* expansion is performed on whole CB units; which is then infused alongside with an un-manipulated CB [29], [31], [32], [33]. New expansion methods attempt to develop serum-free culture systems [34], [35], optimize culture conditions [36], [37], use histone deacetylases [38] which promote self-renewal, use glycogen synthase kinase (GSK)-3 inhibitors [39] which maintain HSC pluripotency, or use tetraethylenepentamine (TEPA) [40] which modulates the proliferation and differentiation of primitive hematopoietic progenitors. Moreover, another new system currently being used is the DIDECO “Pluricell System” which is a commercially available closed device composed of an expansion chamber and a kit of certified reagents that allow haematopoietic stem cell expansion [41]. Recently, de Lima *et al* [33] has shown that TEPA (also called StemEx) attenuates the differentiation of *ex vivo* cultured HSCs resulting in preferential expansion of early progenitors. In a recent phase I/II trial on CD133+ CB HSCs cultured in media containing TEPA, together with SCF, FLT-3 ligand, IL-6, and TPO, nine out of ten patients showed engraftment with an increase of CD34+ cell count, better median time to neutrophil, better platelet engraftment, and no cases of grades 3–4 acute GvHD. Indeed, it has been shown that time to neutrophil engraftment is strongly associated with CD34+ cell dose.

By mimicking the niche, BME were added to our cells prior to their implantation into the mice, which proved to produce an increase in the number of differentiated progenitors and an increase in the engraftment levels. As compared to other currently tested expansion methods, one advantage of our system is that BME were used without the addition of any cytokines, which significantly reduces the cost. A further advantage of our system is that the expansion of cells, after incubation with BME, was not selective of the short-term (low quality) HSCs at the expense of the long-term (high quality) reconstituting HSC. This statement is supported by the engraftment after 6 weeks. However, expansion of the short-term progenitors is also clinically useful since it helps in initial recovery (short-term hematopoietic reconstitution) and especially when combined with un-manipulated CB. However, in order to clearly confirm self-renewal and long term reconstitution from HSCs (LT-SRC), further studies need to be performed using secondary transplantation or engraftment at 12–18 weeks. A third advantage of our system is that cells were cultured for only 1 week for MNCs (or approx. 13 days in the case of CD34+ cells) instead of the 2–3 weeks period used by others [23], [26], [33], which minimizes the risks associated with long term tissue culture such as genetic instability or bacterial and fungal contamination.

These results establish the basis for exploring the use of BME in the expansion of CB progenitors and/or HSC prior to HSCT. It is conceivable that a mixture of an *ex-vivo* expanded/differentiated pool with an untreated pool will result in improved short term and long term reconstitution. This study stresses the importance of the mechanical structure and soluble mediators present in the surrounding niche for the proper activity and differentiation of stem cells.
